# Measuring student satisfaction as the first assessment of the Program of Combined Studies in Medicine, an MD/PhD-like program of the Faculty of Medicine, National Autonomous University of Mexico

**DOI:** 10.1186/s12909-020-02357-1

**Published:** 2020-11-19

**Authors:** Gina Martínez-Flisser, Ana Flisser, Mario Alberto Castro-Guerrero, Tanya Plett-Torres

**Affiliations:** 1 Universidad Anáhuac, Huixquilucan, México; 2 PECEM, Facultad de Medicina, Universidad Nacional Autónoma de México, Avenida Universidad No. 3000, Col. Universidad Nacional Autónoma de México, C.U., Alcaldía Coyoacán, C.P. 04510 Ciudad de México, México; 3 PECEM, Facultad de Medicina, Universidad Nacional Autónoma de México, Ciudad de México, México

**Keywords:** MD/PhD, Medicine, Mentoring, Graduate, Satisfaction, Student support

## Abstract

**Background:**

The Program of Combined Studies in Medicine (PECEM, by its acronym in Spanish) is a program for simultaneous bachelor and doctorate studies (MD/PhD) that enrolls students who show academic excellence and interest in scientific research. The initial doctoral training comprises seven six-month research stays in different laboratories or clinical or computer areas with different high-quality scientific advisors who provide students with a unique experience for their scientific training. Therefore, satisfaction in this stage is decisive for students’ performance and physical and psychological health. The aim of the present study was to administer a questionnaire to measure students’ satisfaction with their research experience as a service-product bundle.

**Methods:**

Students answered an online questionnaire that evaluated three dimensions: perceived quality of the advisor, skills development, and infrastructure and support. Several satisfiers were also evaluated: recommendation of the advisor to peers, fulfillment of student expectations and satisfaction with the program. Correlations were calculated using Fisher’s exact test. The significance was set at *p* < 0.05.

**Results:**

The high quality of the advisor, skills development and guidance during the stay were satisfiers correlated to the students’ recommendation of their advisors to their peers and to the fulfillment of the students’ scientific expectations. Conversely, skills development and infrastructure and support were satisfiers for a good to excellent experience as a PECEM student. A lack of direct interaction with the advisor’s workgroup was related to dissatisfaction.

**Conclusions:**

The nontangible products of the service, such as a positive interaction between the student, the advisor and the advisor’s workgroup as well as support obtained during the research stay, were satisfiers. These data indicate that promoting a fruitful bond between the student and advisor is a priority to ensure the quality of our innovative MD/PhD program.

## Background

The Program of Combined Studies in Medicine (PECEM, by its acronym in Spanish) is a unique MD/PhD curriculum that provides excellent students interested in scientific research the opportunity to become acquainted with biomedical, clinical or sociomedical research. Students start this program by becoming involved in 7 research stays, with a duration of 6 months each, in different laboratories or clinical or computer areas while they complete their bachelor’s studies and before they start their doctoral thesis. Each stay is scheduled after the daily MD curriculum ends (at approximately 3 pm). Students must choose a different advisor, laboratory/area and topic for each stay before they decide on the topic of their doctoral thesis. This structure gives students who were in high school only 2 years before the opportunity to become acquainted with different successful Mexican scientists in an array of different research topics and working environments. Students select their advisors from a list of researchers who work at the National Autonomous University of Mexico (UNAM) or at the National Institutes of Health of Mexico (NIHM) located in Mexico City. Advisors must have published at least 10 original indexed articles in the last 5 years, belong to the National Researchers System of Mexico (SNI, by its acronym in Spanish) or have an updated certification in their medical specialty [1]. The structure of the PECEM is unique and contrasts that of other existing MD/PhD programs, in which students complete their basic studies in medical school, are engaged for 3 to 4 years in full-time research for their PhD thesis and then return to complete their remaining clerkship and medical studies [1, 2]. Other examples are MD/PhD programs in Canada, which mostly involve pre-clerkship training, followed by graduate studies and finally clerkship training, or programs in Europe, where students have 4 years of medical training followed by 3 years of PhD studies and then 2 years of medical school; the exceptions to the characteristic European structure are programs in France and the Netherlands, which have 2 years of parallel medical preparation and research [3, 4].

The purpose of the PECEM is for graduates to become leaders as physician-scientists with very important roles in the translation of research into practice, serving as a bridge between new scientific knowledge and clinical medicine [3, 5]. The literature on this subject suggests that decreased trainee satisfaction during MD/PhD programs might result in attrition and diminish students’ longstanding commitments to sustained research involvement [5]. Thus, quality and improvement of an academic program impacts student satisfaction, which affects a student’s performance and physical and psychological health and correlates with academic activity-related factors [6–9]. In this sense, information provided by students on the presently studied dimensions will help the PECEM and other PhD programs develop new strategies for continuous improvement that take into account aspects that promote an adequate environment to ensure that students feel satisfied and fulfilled and recommend the program.

Student satisfaction, understood as the result of experience, service and facilities, was first measured in the university context using common satisfaction frameworks. Later, higher education models were developed that focused mainly on variables that do not apply to the PECEM experience, such as the classroom environment and lectures, campus climate, social life and teaching staff [10]. Negative quality models have been shown to be helpful for higher education organizations to respond to incidents that lead to dissatisfaction. It has been found that faculty members are consistently important for student withdrawal/persistence decisions, with faculty mentoring programs being positively correlated with academic performance and lower drop-out rates. Likewise, it has been observed that although the institution’s physical facilities are not of significant importance for student satisfaction, the quality of feedback and lecturer-student relationships are key aspects related to satisfaction [11–16].

Recent models for evaluating student satisfaction have focused on the effectiveness of academic advising, instructional assistance, financial aid, and registration; climate and campus life, support services, safety and security; and recruiting, excellence in service and student centeredness [17]. Such models have grouped similar variables in dimensions such as physical goods, facilitating goods, and implicit and explicit services [14]. Other models have suggested that student satisfaction in higher education is determined by the institute’s image, student expectations, the perceived value, and the technical or functional quality [18]. Student satisfaction has also been measured based on the assessment of faculty credentials, financial performance, and the achievement of learner outcomes; comparison with other programs; benchmarking and questionnaires; and most recently, by hybrid models of both satisfaction and facilities [10, 19–21]. Recently, most of the focus of studies on PhD programs has been on students’ academic progress (i.e., publications and conferences); although there are no completely accurate ways to assess satisfaction from a doctoral student’s perspective, examining doctoral student perspectives is an opportunity that can lead to positive changes at the systemic and institutional levels [22]. Taking into account the models used to evaluate student satisfaction, we created a framework to better understand which dimensions of the unique experience obtained by rotating through different research areas influence the satisfaction of PECEM students and motivate them to continue their careers to become successful physician-scientists [23].

Based on the literature review, we hypothesized that the most prevalent satisfiers would be those related to the quality of the advisor, while dissatisfaction would be more frequently related to the infrastructure and support during the research stay. Since our objective was to measure student satisfaction in a unique non-classroom environment, we created a questionnaire to assess students’ PECEM research experience as a service-product bundle (a tangible product of service delivery) outside the classroom setting. The questionnaire consisted of three dimensions: 1) the physical or facilitating goods, 2) the perceived service provided (explicit service), and 3) the psychological service (implicit service) [14, 24]. The results constitute the first attempt to evaluate students’ opinions during their development of professional skills outside the classroom and will allow us to make adjustments to the way the research stays are carried out, enhance the quality of advisors and increase the usefulness of the development of real-world skills that can be translated in the job market.

## Methods

Student satisfaction with their research stays and the related attributes were evaluated with an 18-item online survey (administered via Google Forms). Only information from students and experts who agreed to participate in the study and provided informed consent was included in the analysis. The participants’ names were kept confidential. The project was registered at the UNAM’s Faculty of Medicine Research and Ethics Board (FM/DI/109/2018).

The items included in the questionnaire concerned aspects that the program has addressed since its establishment, as well as those described in a previous study [25]. These aspects were categorized under the following dimensions: attributes related to the quality of the advisor, skills development, and infrastructure and support during the research stay. All items included response scales ranging from the most positive to the most negative perception. The selected dimensions were as follows.

1. The perceived quality of the advisor (the psychological service) comprised 5 variables based on the student’s perception of the advisor’s a) knowledge of the field, b) attitude towards science in Mexico, c) willingness to share experiences and knowledge, d) feedback to the student, and e) amount of time spent directly with the student. The students’ responses were key to evaluating their experience in the PECEM and were based on their relationships with 7 different advisors during the formative period. In this regard, it has been observed that in contrast to an unsatisfactory relationship with the advisor that can lead to withdrawal, a satisfactory relationship influences the quality of education and generates a positive environment, successful socialization and discipline in the institution’s department, and a timely conclusion of the doctoral degree [26–33].
2. Skills development (the perceived service provided) comprised 4 variables: a) scientific training received during the stay, b) usefulness of the research stay, c) diversity of activities during the stay and d) contribution of the stay to the student’s development. The skills developed during research experience are relevant for life and for a student’s marketability to achieve better career prospects and can influence the perceived quality of a program. The perceived quality of a program involves student satisfaction with learned practical skills and the networks created in the professional market (top research fields, groups and institutions) [14, 34, 35].
3. Infrastructure and support (the physical or facilitating goods) comprised 5 variables: a) guidance received from other graduate students in the workgroup, b) guidance received from laboratory technicians, c) facilities used during the research stay, d) supplies provided and e) compatibility of the stay schedule with the student’s activities. These elements, which are provided by administrative and technical staff, are key aspects that optimize experiences in any environment [24, 36].

We explored whether the dimensions described influenced a) the student’s recommendation of the advisors to his/her peers, b) the fulfillment of the student’s scientific expectations and c) the student’s overall experience as a PECEM student. These factors were related to student satisfaction: the questions on the first two factors had two possible answers (yes/no), and the questions on the last factor had scaled responses ranging from the most positive to the most negative perception, which were later grouped into excellent to good or regular to very poor. The descriptions of the satisfaction factors are as follows.

- The student’s recommendation of the advisor to his/her peers: This factor is based on whether the advisor has high levels of interaction with the student, treats him/her as a junior colleague by offering accessibility and connections with faculty members, and promotes regular progress reviews and timely student advancement [26, 28, 31, 37–40].

b) Fulfillment of the student’s scientific expectations: Higher education is a service industry for students (costumers), and institutions have to place greater emphasis on meeting their expectations and needs [7, 41].
c) Experience as a PECEM student: The perceived service quality is defined as a global judgment or attitude related to the superiority of the service and, for an educational service, is what the student feels a program should provide. Satisfaction is the result of the comparison of the expected with the current performance [42, 43].

The responses of content experts (PECEM advisors) to the questionnaire were collected to evaluate its validity in the following aspects: representativeness (the extent to which the items as a whole represent the construct), clarity (the unmistakability of the working of the items) and relevance (the extent to which each item relates to specific aspects of the construct) [29, 44, 45]. The advisors are experts in scientific research, have long held expertise in their fields, and know the key elements necessary to achieve the aim of a research stay and to impact the scientific training of PECEM students.

The relative frequencies were obtained for each item. Fisher’s exact test was used to calculate the correlations between the satisfiers and the items of each dimension. The significance level was set at *p* < 0.05. To identify the satisfiers, the items with the highest frequency of the most positive answers in each dimension were matched with the factors with the highest satisfaction values. The opposite approach was taken to identify dissatisfiers: the items and dimensions were arranged in descending order according to their frequency of negative answers. Information was analyzed using STATA 11.

## Results

### Content validity

Overall, based on a less conservative approach (with at least 80% agreement of the content experts) to assessing the content validity, 94% (17 items) of the questionnaire items on satisfaction were evaluated as clear, 78% (14 items) were evaluated as relevant and 28% (5 items) were evaluated as representative (Table 1) [45]. One item included in the questionnaire was not incorporated in the analysis because it was not considered representative of the students’ satisfaction; it was related only to the program’s postgraduate enrollment processes.

**Table 1:** Validity results of the satisfaction questionnaire

Dimension	Item	Aspects (6 experts)											
		Clarity				Relevance				Representativeness			
		Yes		No		Yes		No		Yes		No	
		n	%	n	%	n	%	n	%	n	%	n	%
**Perceived quality of the advisor**	**I1: The level of the advisor’s knowledge of the field was:**	6	100	0	0	6	100	0	0	4	67	2	33
	*Very limited*												
	*Limited*												
	*Regular*												
	*Broad*												
	*Very broad*												
	**I2: The advisor’s attitude towards science in Mexico was:**	5	83	1	17	4	67	2	33	2	33	4	67
	*Very negative*												
	*Negative*												
	*Neutral*												
	*Positive*												
	*Very positive*												
	**I3: The advisor’s willingness to share experiences and knowledge with you was:**	6	100	0	0	6	100	0	0	5	83	1	17
	*Very poor*												
	*Poor*												
	*Regular*												
	*Good*												
	*Excellent*												
	**I4: How often did you receive feedback from your advisor?**	6	100	0	0	6	100	0	0	5	83	1	17
	*Never*												
	*Sporadically*												
	*Frequently*												
	*Continuously*												
	**I5: The amount of time the advisor spent directly with you was:**	6	100	0	0	6	100	0	0	4	67	2	33
	*None*												
	*A little*												
	*Some*												
	*A lot*												
**Skills development**	**I7: The scientific training you received during the stay was:**	5	83	1	17	6	100	0	0	4	67	2	33
	*Very poor*												
	*Poor*												
	*Regular*												
	*Good*												
	*Excellent*												
	**I8: The research stay that you completed was:**	5	83	1	17	6	100	0	0	4	67	2	33
	*Useless*												
	*A bit useful*												
	*Useful*												
	*Very useful*												
	**I9: The activities you did during the stay were:**	4	67	2	33	3	50	3	50	3	50	3	50
	*Not diverse*												
	*A bit diverse*												
	*Diverse*												
	*Very diverse*												
	**I14: The contribution of the stay to your development was:**	6	100	0	0	6	100	0	0	5	83	1	17
	*Very poor*												
	*Poor*												
	*Regular*												
	*Good*												
	*Excellent*												
**Infrastructure and support**	**I10: The guidance that you received from the graduate students was:**	6	100	0	0	5	83	1	17	4	67	2	33
	*I didn’t receive any guidance*												
	*Very poor*												
	*Poor*												
	*Regular*												
	*Good*												
	*Excellent*												
	**I11: The guidance that you received from the laboratory technician was:**	6	100	0	0	3	50	3	50	3	50	3	50
	*I didn’t receive any guidance*												
	*Very poor*												
	*Poor*												
	*Regular*												
	*Good*												
	*Excellent*												
	**I12: The facilities used during the research stay were:**	5	83	1	17	6	100	0	0	4	67	2	33
	*Very poor*												
	*Poor*												
	*Regular*												
	*Good*												
	*Excellent*												
	**I13: The supplies that you had during the research stay were:**	5	83	1	17	6	100	0	0	4	67	2	33
	*I don’t know*												
	*Null*												
	*Insufficient*												
	*Sufficient*												
	*Excessive*												
	**I6: The compatibility of the stay schedule with your studies was:**	6	100	0	0	5	83	1	17	4	67	2	33
	*Very poor*												
	*Poor*												
	*Regular*												
	*Good*												
	*Excellent*												
**Factors**	**I15: Did the research stay fulfill your scientific training expectations?**	5	83	1	17	5	83	1	17	4	67	2	33
	*Yes*												
	*No*												
	**I16: Will you recommend the advisor to other students?**	6	100	0	0	6	100	0	0	5	83	1	17
	*Yes*												
	*No*												
	**I17: The process of enrollment in the postgraduate program is:**	6	100	0	0	4	67	2	33	3	50	3	50
	*Very complex*												
	*Complex*												
	*Easy*												
	*Very easy*												
	**I18: Your experience as a PECEM student is:**	6	100	0	0	6	100	0	0	5	83	1	17
	*Excellent*												
	*Good*												
	*Regular*												
	*Poor*												
	*Very poor*												

*I* Item

### Description of the population

The total number of research stays in the PECEM as of 2019 was 477, which reflected the students’ time spent in the program: the first, second, third, fourth and fifth generation (9, 11, 10, 13 and 11 students, respectively) had 7 six-month stays per student; the sixth generation (10 students) had 5 six-month stays per student; the seventh generation (11 students) had 3 six-month stays per student; and the eighth generation (16 students) had 1 six-month stay per student. A total of 262 of the 477 stays (55%) were evaluated, and these stays involved 152 different advisors. Each generation of students completed the questionnaire according to the number of research stays that they had to evaluate; 35% of the stays were evaluated by first-generation students (22/63), 55% of the stays were evaluated by second-generation students (42/77), 31% of the stays were evaluated by third-generation students (22/70), 57% of the stays were evaluated by fourth-generation students (52/91), 75% of the stays were evaluated by fifth-generation students (58/77), 72% of the stays were evaluated by sixth-generation students (36/50), 42% of the stays were evaluated by seventh-generation students (14/33) and 100% of the stays were evaluated by eight-generation students (16/16) (data not in tables).

### Distribution of the stay characteristics, dimensions and satisfiers

Biomedical stays were the most frequent (56%; 146), and the National Institutes of Health were the most commonly reported institutions where stays were conducted (60%; 156), with The National Institute of Medical Sciences and Nutrition being the institution most requested by students (34%; 88) (data not in tables). An excellent to good experience as a PECEM student (94%; 245) was the most prominent satisfier, followed by fulfillment of scientific expectations (88%; 230) and 87% (228) recommendation of the advisor (Table 2). The most frequent student perceptions of the advisors included an ample level of knowledge (84%; 221), a very positive attitude towards science (65%; 169) and an excellent willingness to share experiences and knowledge (62%; 162). For more than half of the stays, the students reported that the research stay was very useful (66%; 172), that the stay made an excellent contribution to their development (62%; 162) and that they received excellent training (53%; 138). However, the students reported that only 37% (98) of the stays had very diverse activities. The PECEM is an excellent program, but student perceptions of low feedback from the advisor and insufficient time spent with the student (48%; 125 and 47%; 124, respectively) were of concern since the advisors and stays are the backbone of medical students’ engagement and motivation in scientific research.

**Table 2:** Distribution of dimensions according to the satisfiers

Dimensions	Characteristic	Factors														
		Recommendation of the advisor					Fulfillment of scientific expectations					Experience as a PECEM student				
	***n*** = 262	Yes		No		p*	Yes		No		p*	Excellent-good		Regular-very poor		p*
		n	%	n	%		n	%	n	%		n	%	n	%	
		228	87	34	13		230	88	32	12		245	94	17	6	
**Perceived quality of the advisor**	**The level of the advisor’s knowledge of the field was:**															
	*Very limited* (2)	0	0	2	6	**< 0.001**	0	0	2	6	**< 0.001**	2	1	0	0	0.058
	*Limited (0)*	0	0	0	0		0	0	0	0		0	0	0	0	
	*Regular* (6)	5	2	1	3		4	2	2	6		5	2	1	6	
	*Broad* (33)	20	9	13	38		24	10	9	28		28	11	5	29	
	*Very broad (221)*	203	89	18	53		202	88	19	60		210	86	11	65	
	**The advisor’s attitude towards science in Mexico was:**															
	*Very negative* (1)	0	0	1	3	**< 0.001**	0	0	1	3	**0.002**	0	0	1	6	0.086
	*Negative* (4)	3	1	1	3		3	1	1	3		4	2	0	0	
	*Neutral* (16)	4	2	12	35		10	4	6	19		14	6	2	12	
	*Positive (72)*	63	28	9	27		62	27	10	31		67	27	5	29	
	*Very positive (169)*	158	69	11	32		155	67	14	44		160	65	9	53	
	**The advisor’s willingness to share experiences and knowledge with you was:**															
	*Very poor* (6)	0	0	6	18	**< 0.001**	1	1	5	16	**< 0.001**	5	2	1	6	**0.043**
	*Poor* (4)	0	0	4	12		1	1	3	9		2	1	2	12	
	*Regular* (24)	15	7	9	26		17	7	7	22		23	9	1	6	
	*Good (66)*	58	25	8	23		56	24	10	31		62	25	4	23	
	*Excellent (162)*	155	68	7	21		155	67	7	22		153	63	9	53	
	**How often did you receive feedback from your advisor?**															
	*Never* (8)	3	1	5	15	**< 0.001**	4	2	4	12	**< 0.001**	7	3	1	6	0.368
	*Sporadically* (49)	31	14	18	53		34	15	15	47		44	18	5	29	
	*Frequently (80)*	75	33	5	15		72	31	8	25		75	31	5	29	
	*Continuously (125)*	119	52	6	17		120	52	5	16		119	48	6	36	
	**The amount of time the advisor spent directly with you was:**															
	*None* (9)	3	1	6	18	**< 0.001**	4	2	5	16	**< 0.001**	7	3	2	12	**0.015**
	*A little* (31)	15	7	16	47		20	8	11	34		26	10	5	29	
	*Some (98)*	91	40	7	20		87	38	11	34		95	39	3	18	
	*A lot (124)*	119	52	5	15		119	52	5	16		117	48	7	41	
**Skills development**	**The scientific training you received during the stay was:**															
	*Very poor* (6)	0	0	6	18	**< 0.001**	0	0	6	19	**< 0.001**	5	2	1	6	**0.002**
	*Poor* (3)	1	1	2	6		1	1	2	6		2	1	1	6	
	*Regular* (18)	9	4	9	26		5	2	13	41		13	5	5	29	
	*Good (97)*	85	37	12	35		87	38	10	31		93	38	4	24	
	*Excellent (138)*	133	58	5	15		137	59	1	3		132	54	6	35	
	**The research stay that you completed was:**															
	*Useless* (5)	0	0	5	15	**< 0.001**	0	0	5	16	**< 0.001**	5	2	0	0	**0.001**
	*A bit useful* (18)	8	3	10	29		5	2	13	41		12	5	6	35	
	*Useful (67)*	54	24	13	38		55	24	12	37		63	26	4	24	
	*Very useful (172)*	166	73	6	18		170	74	2	6		165	67	7	41	
	**The activities you did during the stay were:**															
	*Not diverse* (6)	0	0	6	18	**< 0.001**	1	1	5	16	**< 0.001**	6	2	0	0	**0.007**
	*A bit diverse* (47)	36	16	11	32		30	13	17	53		38	16	9	52	
	*Diverse (111)*	98	43	13	38		104	45	7	22		107	44	4	24	
	*Very diverse (98)*	94	41	4	12		95	41	3	9		94	38	4	24	
	**The contribution of the stay in your development was:**															
	*Very poor* (4)	0	0	4	12	**< 0.001**	0	0	4	12	**< 0.001**	3	1	1	6	**< 0.001**
	*Poor* (3)	2	1	1	3		0	0	3	9		1	1	2	12	
	*Regular* (12)	2	1	10	29		0	0	12	38		7	3	5	29	
	*Good (81)*	68	30	13	38		69	30	12	38		79	32	2	12	
	*Excellent (162)*	156	68	6	18		161	70	1	3		155	63	7	41	
**Infrastructure and support**	**The guidance that you received from the graduate students was:**															
	*I didn’t receive any guidance* (42)	37	16	5	15	**0.001**	35	15	7	22	**< 0.001**	39	16	3	18	0.073
	*Very poor* (2)	0	0	2	6		0	0	2	6		1	1	1	6	
	*Poor* (1)	1	1	0	0		1	1	0	0		1	1	0	0	
	*Regular* (17)	11	5	6	18		10	4	7	22		14	5	3	18	
	*Good (64)*	53	23	11	32		51	22	13	41		62	25	2	11	
	*Excellent (136)*	126	55	10	29		133	58	3	9		128	52	8	47	
	**The guidance that you received from the laboratory technician was:**															
	*I didn’t receive any guidance (91)*	77	34	14	41	**0.011**	78	34	13	41	**< 0.001**	84	34	7	41	0.578
	*Very poor* (3)	2	1	1	3		3	1	0	0		3	1	0	0	
	*Poor* (3)	2	1	1	3		1	1	2	6		3	1	0	0	
	*Regular* (13)	11	5	2	6		10	4	3	9		11	5	2	11	
	*Good (60)*	48	21	12	35		47	20	13	41		56	23	4	24	
	*Excellent (92)*	88	38	4	12		91	40	1	3		88	36	4	24	
	**The facilities used during the research stay were:**															
	*Very poor* (1)	0	0	1	3	**0.012**	1	1	0	0	**0.015**	1	1	0	0	0.769
	*Poor* (4)	4	2	0	0		3	1	1	3		4	2	0	0	
	*Regular* (18)	13	6	5	15		13	5	5	16		17	7	1	6	
	*Good (104)*	87	38	17	50		87	38	17	53		99	40	5	29	
	*Excellent (135)*	124	54	11	32		126	55	9	28		124	50	11	65	
	**The supplies that you had during the research stay were:**															
	*I don’t know* (9)	6	3	3	9	**0.001**	3	1	6	19	**< 0.001**	5	2	4	24	**< 0.001**
	*Null* (2)	0	0	2	6		1	1	1	3		2	1	0	0	
	*Insufficient* (5)	3	1	2	6		4	2	1	3		4	2	1	6	
	*Sufficient (208)*	183	80	25	73		187	81	21	66		201	82	7	41	
	*Excessive* (38)	36	16	2	6		35	15	3	9		33	13	5	29	
	**The compatibility of the stay schedule with your studies was:**															
	*Very poor* (5)	3	1	2	6	**< 0.001**	3	2	2	6	**< 0.001**	3	1	2	12	**0.011**
	*Poor* (16)	14	6	2	6		12	5	4	13		13	5	3	18	
	*Regular (57)*	42	19	15	44		42	18	15	47		54	22	3	18	
	*Good (82)*	71	31	11	32		74	32	8	25		80	33	2	11	
	*Excellent (102)*	98	43	4	12		99	43	3	9		95	39	7	41	

*p value for Fisher’s exact test

While the students reported receiving no guidance from graduate students in the advisor’s workgroup for some of the stays (16%; 42), for more than half of the stays, the students indicated that they received excellent guidance from graduate students (52%; 136) or laboratory technicians (55%; 75). In contrast, it was worrying that for other stays, the students reported a lack of guidance from laboratory technicians (35%; 91), and it was even more worrying the lack of guidance from graduate students and from laboratory technicians in 33 research stays (13%) (data not in tables). For more than half of the stays, the students reported that there were excellent facilities (52%; 135), and for most of the stays, the students indicated that there were sufficient supplies (79%; 208); however, the schedules of only 39% (102) of the stays were considered to have excellent compatibility with the students’ activities.

### Recommendation of the advisor

Significant correlations were found between students’ willingness to recommend their advisors to their peers and all the studied dimensions (*p* < 0.05, Table 2). The dimension with the highest frequency of positive answers was the perceived quality of the advisor (intangible attribute) regarding his/her ample knowledge (89%; 203), very positive attitude towards science in Mexico (69%; 158) and excellent willingness to share knowledge (68%; 155). Real-world skills (skills development) also had a high frequency of positive answers among the students who reported that they would recommend their advisors: 73% (166) of the stays evaluated by students who would recommend their advisors were considered very useful, and 68% (156) were perceived to have an excellent contribution to the students’ academic development. Together with the diversity of activities during the stay (41%; 94), the frequency of positive answers for items related to infrastructure and support (tangible attributes) was less than 60% among students who reported that they would recommend their advisors. These findings were similar to what we expected: rather than the tangible attributes of the research stay, intangible attributes, such as close contact with the advisor and the development of real-world skills, tended to have a higher influence on the students’ willingness to recommend an advisor.

### Fulfillment of scientific expectations

Significant correlations were also found between the fulfillment of scientific expectations and all the studied dimensions (*p* < 0.05, Table 2). The perceived quality of the advisor was also the most frequently reported dimension, and the advisor’s attributes, such as his/her ample knowledge (88%; 202), very positive attitude towards science in Mexico or excellent willingness to share experiences and knowledge (67%; 155), were also frequent. A lower percentage of stays were reported to involve the advisor spending a large amount of time with students or providing continuous feedback (52%; 120 and 119, respectively). However, among the stays for which students reported that their expectations were fulfilled and that the advisor had a very positive attitude towards science (155), there was a high frequency of responses indicating the advisor’s excellent willingness to share experiences and knowledge (119) and provide continuous feedback (117) (77 and 75%, respectively, data not in table). Similarly, the perceptions that the stay was very useful (74%; 170) and that it made an excellent contribution to the students’ development (70%; 161) were the most frequently cited real-world skills. On the other hand, interestingly, the highest positive evaluation for infrastructure and support was sufficient supplies (81%; 187), while attributes such as excellent guidance from graduate students in the advisor’s workgroup (58%; 133), excellent facilities (55%; 126), the compatibility of the stay schedule with the student’s activities (43%; 99), and guidance from the laboratory technician (40%; 91) had lower frequencies of positive evaluations. Intriguingly, we found that even though for some of the stays, the students reported that they never received guidance from graduate students (15%; 35) and/or laboratory technicians (34%, 78), they considered that these research stays fulfilled their scientific expectations. These observations suggest that, similar to what was found among students who would recommend their advisors, these students perceived that their scientific expectations had been fulfilled mostly when they had close contact with their advisors and when they experienced positive development of their skills.

### Experience as a PECEM student

Unlike other factors, experience as a PECEM student was significantly correlated with half of the studied dimensions (*p* < 0.05, Table 2). The advisor’s ample level of knowledge and very positive attitude towards science in Mexico had borderline significance, and the only attributes of the perceived quality of the advisor that showed significance were the willingness to share experiences and the amount of time spent with the student, with frequencies of 63% (153) for excellent willingness to share experiences and 48% (117) for a large amount of time spent with the student, respectively. All the attributes of skills development were significantly correlated with good to excellent experience as a PECEM student, with scientific training, diversity of activities, usefulness of the stay and excellent contribution to the student’s development having frequencies of positive perceptions above 80%. Additionally, the aspects of infrastructure and support that showed significant correlations with good to excellent experience as a PECEM student were the availability of supplies during the research stay (sufficient, 82%; 201) and the compatibility of the stay schedule with the student’s activities (excellent, 39%; 95). These results imply the influence of close contact with the advisor on the perception of a positive experience as a PECEM student, but the most important aspects were the development of real-world skills and the infrastructure and support during the research stay.

### Dimensions and items associated with the highest satisfaction

The perceived quality of the advisor was the dimension that was most correlated with student satisfaction. This was observed for recommendation of the advisor for the stays of other students and the fulfillment of scientific expectations (Table 3). As we already mentioned, this dimension comprised intangible attributes of close contact with the advisor. The frequency with which these attributes were reported were, in descending order, ample knowledge in the field, very positive attitudes towards science in Mexico, excellent willingness to share experiences, a large amount of time spent with the student and continuous feedback. On the other hand, contrary to what we expected, we found that real-world skills (skills development) was the most frequent dimension reported for a good to excellent experience as a PECEM student, followed by (in descending order of frequency): very useful stay, excellent contribution to the student’s development, scientific training and very diverse activities. Interestingly, for this satisfier, the second most frequently cited dimension was infrastructure and support, which represent tangible attributes, and surprisingly, the dimension with the lowest influence was the perceived quality of the advisor.

**Table 3:** Dimensions and their items as aspects of satisfaction or dissatisfaction

	Satisfaction			
**Dimensions**	**No.**	**Recommendation of the advisor**	**Fulfillment of scientific expectations**	**Excellent to good experience as a PECEM student**
	**1**	**Perceived quality of the advisor**		**Skills development**
		1. Knowledge*		1. Usefulness*
		2. Attitude*		2. Contribution*
		3. Willingness*		3. Training*
		4. Time spent*		4. Diversity*
		5. Feedback*		
	**2**	**Skills development**		**Infrastructure and support**
		1. Usefulness*		*1. Guidance from the graduate students*
		2. Contribution*		*2. Facilities*
		3. Training*		3. Compatibility*
		4. Diversity*		*4. Guidance from the laboratory technician*
				5. Supplies*
	**3**	**Infrastructure and support**		**Perceived quality of the advisor**
		1. Guidance from the graduate students*		*1. Knowledge*
		2. Facilities*		*2. Attitude*
		3. Compatibility*		3. Willingness*
		4. Guidance from the laboratory technician*		4. Time spent*
		5. Supplies*		*5. Feedback*
	**Dissatisfaction**			
**Dimensions**	**No.**	**No recommendation of the advisor**	**Lack of fulfillment of scientific expectations**	**Regular to very poor experience as a PECEM student**
	**1**	**Infrastructure and support**		
		1. Guidance from the laboratory technician**		
		2. Guidance from the graduate students*		2. Supplies*
		3. Supplies*		*3. Guidance from the graduate students*
		4. Compatibility*		
		5. Facilities***		
	**2**	**Perceived quality of the advisor**		
		1. Time spent*	1. Willingness*	1. Time spent*
		2. Willingness*	2. Time spent*	*2. Feedback*
		3. Feedback*		3. Willingness*
		4. Knowledge*		*4. Attitude*
		5. Attitude*		*5. Knowledge§*
	**3**	**Skills development**		
		1. Training*		1. Contribution*
		2. Diversity*	2. Usefulness*	2. Training*
		3. Usefulness*	3. Diversity*	3. Usefulness§*
		4. Contribution*		4. Diversity§*

*Items with *p* < 0.05

**p < 0.05 for no recommendation of the advisor and lack of fulfillment of scientific expectations

***p < 0.05 for no recommendation of the advisor and lack of fulfillment of scientific expectations; lack of fulfillment of scientific expectations and regular to very poor experience as a PECEM student were not selected by students

§ No students selected the item

### Dimensions and items influencing dissatisfaction

As expected, dissatisfaction was indicated by an unwillingness to recommend the advisor, a lack of fulfillment of scientific expectations and/or regular to very poor experience as a PECEM student, and it was influenced by tangible attributes such as the lowest availability of infrastructure and support and mostly low interaction with the advisor’s workgroup, such as a lack of guidance from postgraduate students or laboratory technicians (Table 3). Other items, such as the advisor’s lack of knowledge, insufficient supplies available during the stay, very poor compatibility of the stay schedule with the student’s activities and very poor facilities, were also related to dissatisfaction. The next most frequently cited attributes were linked to the perceived quality of the advisor, generally reflecting low interaction with the student (lack of time spent with the student, very poor willingness to share experiences and a lack of feedback). The dimension with the lowest influence on dissatisfaction was skills development, including very poor training, a useless stay and a very poor contribution to the student’s scientific development.

## Discussion

As expected, close contact between the advisor and the PECEM student seems to be a very important element for satisfaction; thus, this interaction must be highlighted at the beginning of each stay with each advisor. In this regard, the advisor’s ample knowledge in his/her research field, very positive attitude towards science in Mexico and excellent willingness to share experiences and knowledge were the most frequent attributes cited by students who reported that they would recommend their advisors and that the stay fulfilled their scientific expectations.

The relationship that doctoral students develop with advisors is one of the most important aspects of their graduate education [46]. In the unique structure of the PECEM, students relate to 10 advisors, 7 whom they work with during their six-month stay periods during their undergraduate training and 3 who are members of their doctoral committees that follow and evaluate them and their thesis projects, which last 3 to 4 years. The doctoral committee includes the main thesis advisor and two other members. Reports have shown that the prevalence of student satisfaction is equal to or higher than 50% [47]. In our study, the prevalence of student satisfaction with the three factors evaluated was high: there was a 94% prevalence of good to excellent experience as a PECEM student, an 88% prevalence of the perception that the stay fulfilled the student’s scientific expectations and an 87% prevalence of student recommendation of the advisor. Considering the high frequency with which the students recommended the advisor that they initially selected, we infer that students’ experience of participating in 7 research stays in their first years at the PECEM is appropriate and that other graduate programs should include in their curricula the PECEM experience, offering various research areas for each student. Likewise, this experience allows the list of advisors participating in the program to be updated.

The institution’s status has been found to be an influential factor of the significant relationship between student satisfaction and teaching quality: students are satisfied if they receive good teaching in a trustworthy place [48, 49]. To ensure that student expectations are met (satisfaction), there have been multiple national surveys in developed countries indicating that satisfied students are more likely to be those who have a curriculum that aligns perfectly with their expectations and subsequent specific job world [19]. These observations are similar to what we identified in our study: the dimension that was most frequently reported by the participants who had a good to excellent experience as a PECEM student was the dimension that integrated real-world skills, such as the high usefulness of the stay and the excellent contribution of the stay to the student’s development. Importantly, in each research stay, the student is expected to develop skills in scientific research and to acquire experiences in the field for their future practice.

We have mentioned that, as expected, the perception of the high quality of the advisor was frequent in satisfied students, and surprisingly, the excellent development of skills was also commonly reported by these students. On the other hand, we found that poor support and attributes of poor infrastructure during the research stay were frequently cited by students with low satisfaction. Although we expected this finding, the identification of a lack of guidance by the advisor’s workgroup as an important factor for student dissatisfaction was interesting; this finding reinforces the fact that close contact with the student is important for a positive experience during the research stay. One of the points that we initially considered important in terms of infrastructure and support during the research stay was a lack of compatibility of the undergraduate classes (in the morning) and the stay schedules (starting at 3 pm); however, this attribute seems to be less frequently reported in relation to dissatisfaction.

Student satisfaction is undoubtedly an institutional axis since, in addition to its basic importance, it poses several challenges, including how information is obtained over time, how satisfaction is measured effectively and how satisfaction is promoted [2]. There is much to learn about what makes an excellent student satisfied with his/her experience in a higher education program, especially in programs that are innovative and that are based on the development of real-world skills in research areas. As in the literature review, this study clearly showed that the relationship between the advisor and the student is a cornerstone for satisfaction. Exploring this relationship is crucial in understanding how to manage and improve MD/PhD programs by focusing on the quality of intangible services provided rather than the tangible variables (i.e., budget and facilities). This can be achieved by building better relationships with program advisors and giving them training and tools that facilitate the mentoring process.

The limitations of the present survey are those that are common to cross-sectional studies in terms of the ability to determine only possible correlations and not causality. In addition, we did not include groups of students from other Mexican programs for comparisons since there is no other program like the PECEM in Mexico; thus, the results in this report indicate only the satisfaction of PECEM students. Another limitation is the voluntary nature of the participation of advisors in the validation stage and students in the survey, which implies that those who contributed may have had different interests than those who did not. Finally, since the PECEM is a newly created program, we do not know what the long-term implications of student satisfaction will be.

## Conclusions

Positive interaction between the PECEM student, the advisor and his/her workgroup is a psychological service that complements a perceived service (skills development) to promote satisfaction. These aspects need to be considered when new advisors are integrated into the PECEM and must be reinforced to improve the quality of our innovative MD/PhD program. Therefore, the promotion of a fruitful bond between the student and advisor should be a priority for any doctoral program because this bond was closely related to student satisfaction for all the determining factors in the present study. The proper redirection of budgetary and operational decisions to achieve this goal is imperative for the inclusion of more physician-scientists in the job market, especially currently, when technology and scientific advances are evolving in a fast-paced economy and there is a need to efficiently translate laboratory findings into clinical contexts. A first step is to build better and more lasting relationships between the PECEM staff and advisors to determine the mentoring and practical skills that can help strengthen the bond with students and facilitate their acquisition of real-world skills that can be translated in research positions in the job market.

## Data Availability

The dataset supporting the conclusions of this article is available in the ZENODO repository [DOI: 10.5281/zenodo.3614046 and hyperlink to dataset in https://zenodo.org/record/3614046#.XiYatBe73aY)].

## Abbreviations

PECEM: Plan de Estudios Combinados en Medicina
MD/PhD: Doctor of Medicine and Doctor of Philosophy
MD: Doctor of Medicine
PhD: Doctor of Philosophy
UNAM: Universidad Nacional Autónoma de México
